# A novel approach with tofacitinib for the management of keratoderma blennorrhagicum in reactive arthritis: a case report

**DOI:** 10.3389/fimmu.2024.1399249

**Published:** 2024-07-02

**Authors:** Maierhaba Maitiyaer, Yu Liu, Nueramina Keyimu, Yueqiang Wen, Zhiping Liu, Wenhui Huang, Shuilian Yu

**Affiliations:** ^1^ Department of Rheumatology, The Second Affiliated Hospital, Guangzhou Medical University, Guangzhou, China; ^2^ Department of Clinical Medicine, The First School of Clinical Medicine, Guangzhou Medical University, Guangzhou, China; ^3^ Department of Medical Imaging, The Second School of Clinical Medicine, Guangzhou Medical University, Guangzhou, China; ^4^ Department of Nephrology, The Second Affiliated Hospital, Guangzhou Medical University, Guangzhou, China; ^5^ Ophthalmic Center, The Second Affiliated Hospital, Guangzhou Medical University, Guangzhou, China

**Keywords:** reactive arthritis, Reiter’s syndrome, keratoderma blennorrhagicum, tofacitinib, JAK inhibitors

## Abstract

Reactive arthritis(ReA), a form of arthritis occurring post-infection, manifests with antecedent infection symptoms, arthritis, and extra-articular manifestations, categorizing it as spondyloarthritis. “Keratoderma blennorrhagicum” (characterized by pustular hyperkeratosis on palms and soles, resembling pustular psoriasis) represents the most typical skin manifestation of ReA, occurring in acute or chronic phases. Severe lesions necessitate systemic disease modifying anti-rheumatic drugs (DMARDs) or biologic therapies. This article reports a case of ReA with sacroiliitis and widespread pustular eruptions following a urinary tract infection. Treatment with sulfasalazine and thalidomide significantly improved sacroiliitis, but the skin rash remained persistent and recurring. Subsequent use of adalimumab and secukinumab resulted in worsening skin rash, prompting a switch to tofacitinib, leading to a remarkable improvement in pustular eruptions after 20 days of treatment. This case demonstrates successful application of tofacitinib in treating severe keratoderma blennorrhagicum refractory to conventional DMARDs and biologics, offering insights into JAK inhibition for challenging rheumatic diseases with skin involvement.

## Introduction

Reactive arthritis (ReA), also known as Reiter’s syndrome, is defined as sterile synovitis developing after a distant infection, usually in the genitourinary or gastrointestinal tract (1). This condition was first identified in 1916 by Hans Reiter, who observed a triad of symptoms, including urethritis, conjunctivitis, and arthritis (2).

Keratoderma blennorrhagicum (KB) is a characteristic skin manifestation in patients with ReA. It is a type of dermatitis similar to pustular psoriasis, characterized by hyperkeratotic erythematous lesions. KB appears as clear vesicles on various parts of the body, progressing to macules, papules, nodules, and eventually hyperkeratotic plaques. Fingertips and toes may also be affected. Painful erosions, pustules, and subungual pustular collections can occur, with nail involvement in 20%-30% of patients (2). KB is classified as a type of palmoplantar pustulosis (PPP), along with conditions like psoriasis, palmoplantar pustulosis, acrodermatitis continua, and infantile acropustulosis. While they share sterile pustules on palms and soles, they likely have different causes. Histologically, KB and psoriasis are similar, but KB may have more numerous pustules and extensive hyperkeratosis.

Janus kinases (JAKs) inhibitors are a new class of drugs that can block multiple cytokines by inhibiting JAKs and signal transducers and activators of transcription (STATs). They are administered orally or topically for skin conditions and have shown effectiveness in various disorders. The first oral JAK inhibitors have been approved for rheumatoid arthritis and psoriatic arthritis, with ongoing research investigating their potential use in treating other skin diseases such as alopecia areata, atopic dermatitis, dermatomyositis (3).

This article reports a case of ReA combined with KB, in which the rash significantly improved after treatment with tofacitinib.

## Case report

**Figure 1:**
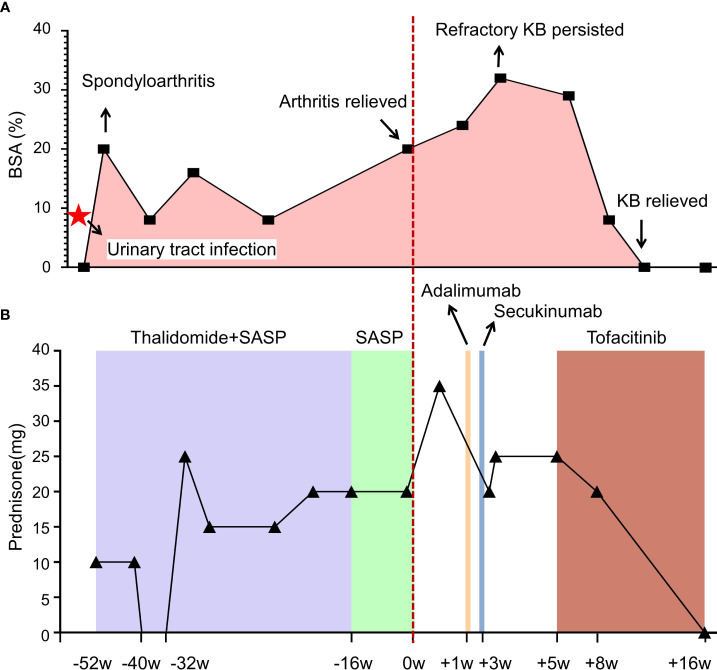
Illustrating clinical progression **(A)** and therapeutic interventions **(B)** in this patient with spondyloarthritis and cutaneous manifestations. Each data point represents a medical consultation (MC), while the body surface area (BSA) delineates the extent of the patient’s cutaneous involvement over time. Initially, prednisone was administered, with dosage adjustments tailored to symptom severity. Thalidomide (50 mg bid) and sulfasalazine (0.5g bid) were prescribed for arthritis management. Despite initial improvement in joint symptoms, the persistent and recurrent skin rash remained unresolved. Subsequent treatment with tumor necrosis factor (TNF)-α inhibitor (adalimumab) and interleukin (IL)-17A inhibitor (secukinumab) failed to alleviate symptoms. However, initiation of tofacitinib (5 mg bid) led to significant improvement, resulting in complete resolution of pustular lesions within 20 days. Follow-up assessments revealed sustained remission, indicating successful disease management.

**Figure 2:**
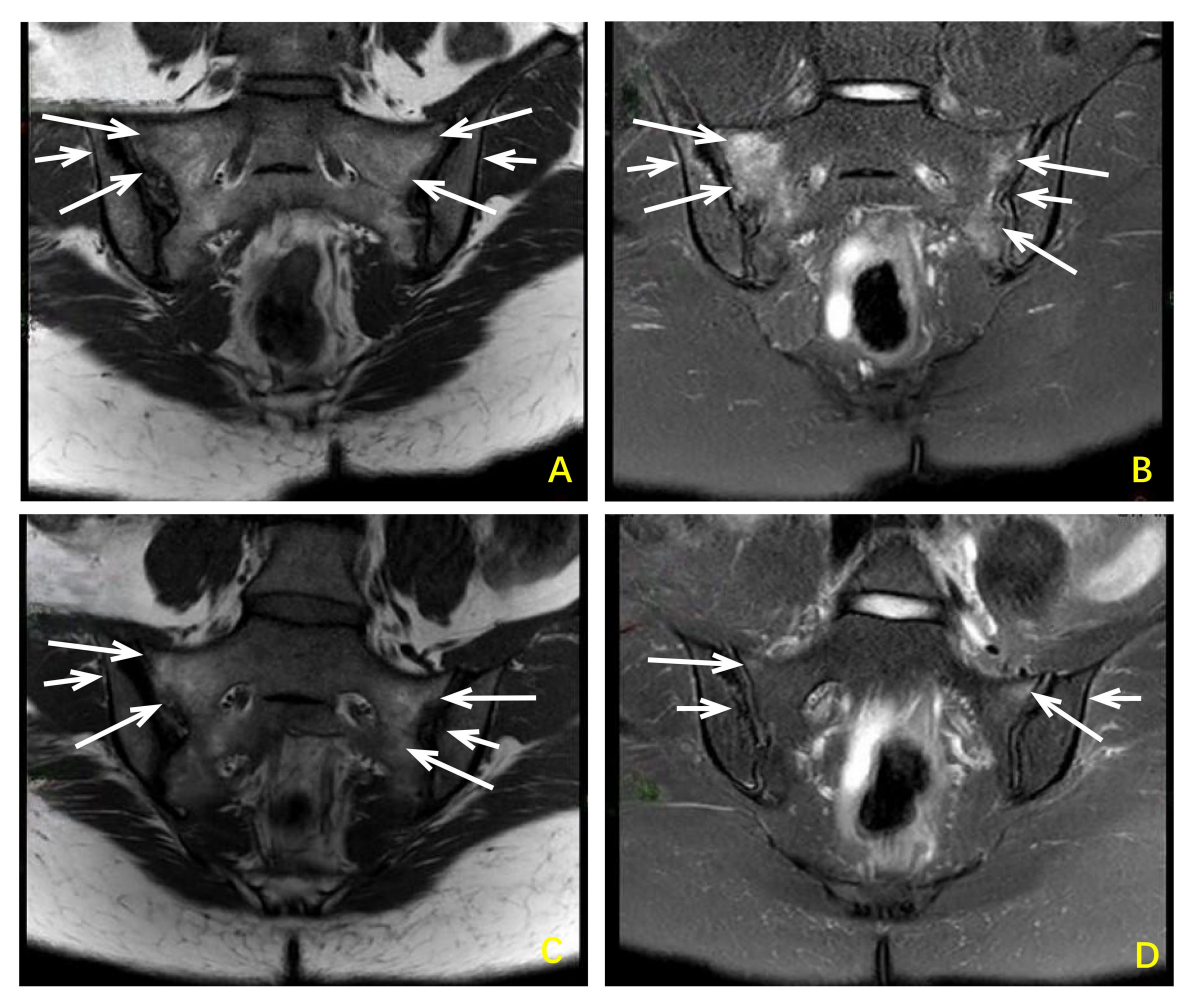
Changes in sacroiliac joint MRI images before and after treatment with sulfasalazine combined with saliduridine in patients with reactive arthritis. Before treatment **(A, B)** Bilateral sacroiliac joints showed irregular patchy slightly low signal intensity on T1-weighted images and slightly high signal intensity on T2-weighted images, with high signal intensity on fat-suppressed T2-weighted images indicating bone marrow edema. Local cortical bone blurring and narrowing of the joint space were observed, and the articular cartilage was indistinct. After treatment **(C, D)** Bilateral sacroiliac joints showed a reduction in the extent of bone marrow edema compared to pre-treatment images. Local cortical bone blurring and narrowing of the joint space remained present, and the articular cartilage remained indistinct.

**Figure 3:**
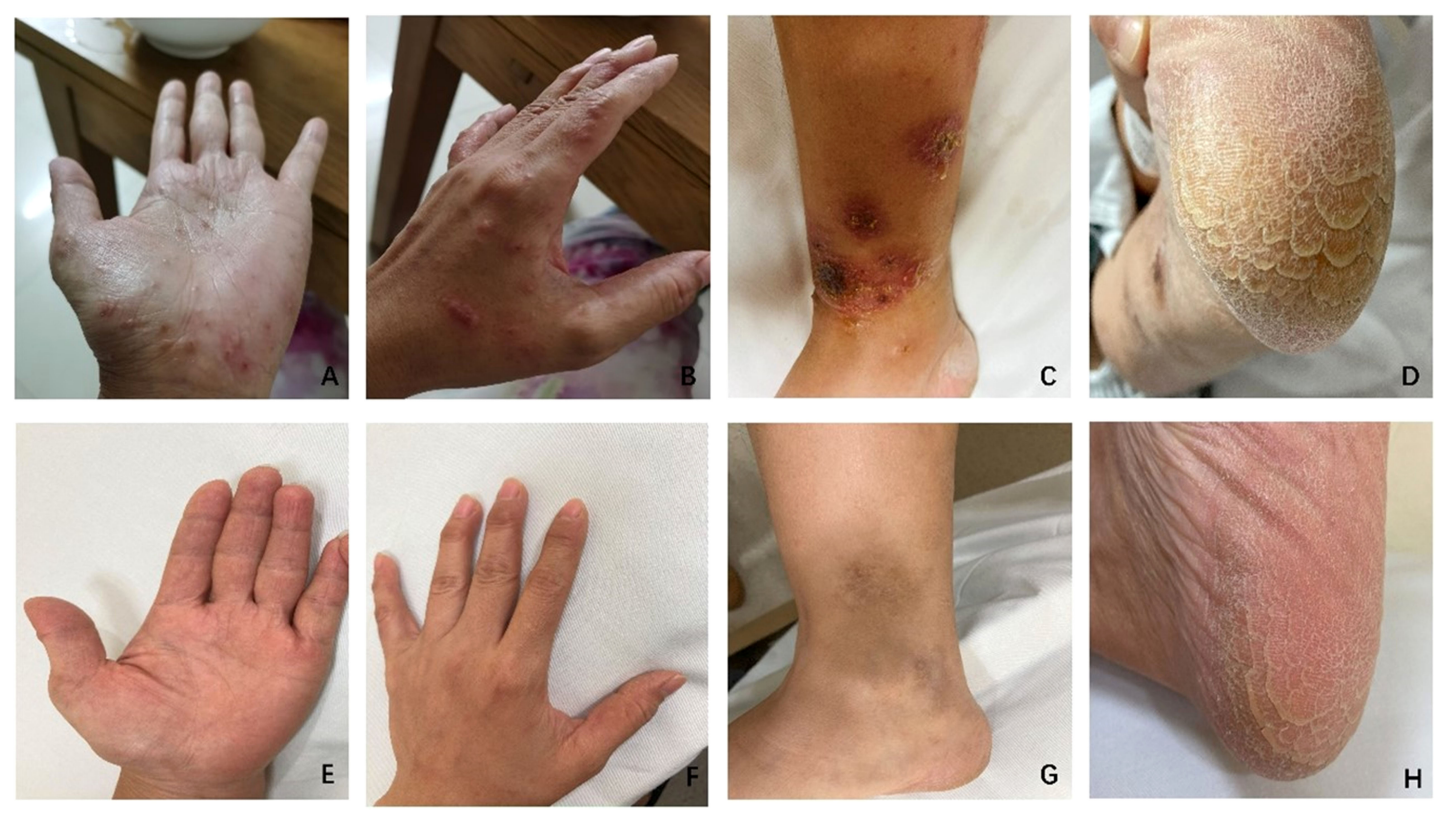
Changes in vesicles and papules on the palmar **(A, E)**, dorsal **(B, F)**, and medial ankle **(C, G)** regions of both hands before and after 20 days of treatment with tofacitinib. Additionally, there was an improvement in the hyperkeratosis of the soles **(D, H)** compared to before treatment. Before treatment **(A-D)** The palmar, dorsal, and medial ankle regions exhibited vesicles and papules. Excessive hyperkeratosis was observed on the soles. After 20 days of treatment **(E-H)** A reduction in size or disappearance of vesicles and papules on the palmar, dorsal, and medial ankle regions was noted. Furthermore, there was an improvement in the hyperkeratosis of the soles.

A 42-year-old female patient was admitted to the hospital with complaints of recurrent pustular eruptions and sacroiliac pain that had been present for one year, with worsening rash over the past 14 days. It is important to note that the patient developed widespread pustular eruptions on her limbs and joint symptoms following a urinary tract infection (**Figure 1A**), with urinalysis revealing elevated white blood cells and positive antibodies against Chlamydia trachomatis. In addition to the skin rash and sacroiliac joint pain, she experienced fever, oral ulcers, conjunctivitis, and perineal pustular lesions. Magnetic resonance imaging (MRI) confirmed bilateral sacroiliitis (**Figures 2A, B**), leading to a diagnosis of ReA. The patient was initially treated with sulfasalazine, thalidomide, and anti-inflammatory drugs, which effectively improved her joint symptoms. However, the persistent and recurrent skin rash did not improve with this treatment. During the recent exacerbation, the patient presented with numerous pustular eruptions on her palms, soles, and lower limbs, accompanied by itching (**Figures 3A–D**). Bacterial cultures and tests for infectious diseases came back negative. Skin biopsies showed psoriasis-like changes without signs of inflammation or vasculitis. Notably, the patient had a history of contact with cats and dogs. While the initial treatment successfully alleviated the joint symptoms and follow-up MRI revealed improvements in the sacroiliitis as well (**Figures 2C, D**), the refractory KB persisted. Due to the patient’s long-term recurrent KB, we attempted to use tumor necrosis factor (TNF) -α inhibitor (adalimumab) and interleukin (IL) -17A inhibitor (secukinumab) (**Figure 1B**). However, the patient’s symptoms did not show significant improvement and instead worsened. Therefore, a therapeutic approach involving a dose of 25 mg prednisone was initiated, leading to crusting of pustules on the lower limbs after 7 days. However, pustular lesions on the palms continued to progress with notable itching. Subsequently, a reduction of prednisone dosage to 20 mg qd, along with the addition of tofacitinib at 5 mg bid (**Figure 1B**), led to the resolution of pustular lesions on both palms and lower limbs after 20 days (**Figures 3E–H**). After three months, prednisone was gradually tapered, and symptoms were managed with tofacitinib monotherapy, with no recurrence observed during subsequent follow-ups.

## Discussion

Although there is currently no universally satisfying disease definition, clinical criteria consider patients with acute symptomatic urethritis, cervicitis, or enteritis occurring in the preceding 1–2 months, coupled with newly onset definitive signs of joint swelling, tenderness, enthesitis, or inflammatory lumbosacral pain, as potential cases of ReA. Extra-articular manifestations encompass conjunctivitis, urogenital tract inflammation, diarrhea, painless mucosal ulcers, KB, nodular erythema, genital lesions, and rare valvular diseases. In the realm of differential diagnosis, ReA must be distinguished from various conditions, including diseases causing acute monoarthritis or oligoarthritis, particularly those related to gastrointestinal or genitourinary symptoms (such as enteric viral infections, inflammatory bowel diseases, or disseminated gonococcal infections), acute suppurative or crystalline arthritis, Behçet’s disease, undifferentiated spondyloarthritis, and other post-infectious arthropathies.

The presented case met diagnostic criteria for ReA based on 1. antecedent urethritis, 2. joint involvement with sacroiliitis and inflammatory lumbosacral pain, and 3. extra-articular manifestations including conjunctivitis, urinary tract infection, painless oral ulcers, perineal pustular lesions, and KB. Regarding differential diagnosis, the initial consideration by the attending physician was Behçet’s disease due to oral ulcers, conjunctivitis, and perineal pustular lesions. However, two biopsies from different sites showed no panniculitis or vasculitis. Additionally, while Behçet typically presents with non-erosive arthritis, the patient’s sacroiliac MRI revealed bone marrow edema, joint surface blurring, and joint space narrowing (**Figures 2A, B**), with a negative HLA-B51 test, thus ruling out Behçet’s disease. Furthermore, axial spondyloarthritis was considered, but the patient’s history of preceding infection, characteristic KB, and negative HLA-B27 test suggest that ReA is the most likely diagnosis.

ReA classified as a type of spondyloarthritis (SpA), is characterized by arthritis that develops following an infection, although the specific pathogen cannot be identified in the affected joints. ReA is relatively uncommon, predominantly affecting young individuals, with both genders susceptible. The global annual incidence ranges from 1 to 30 per 100,000 (4).

KB is a distinctive symptom of skin hyperkeratosis in individuals with ReA, occurring in approximately 5–30% of cases (2). These lesions are commonly observed on plantar skin and may manifest as vesicles, maculopapular rashes, or macules. Nail changes, such as thickening, onycholysis, and subungual keratin deposits, are also observed in individuals with KB (5). Although cutaneous leucocytoclastic vasculitis is considered typical in KB rather than psoriasis pustulosa (PP), clinical and microscopic distinctions between these two diseases are challenging. A distinguishing feature of this disease is the presence of painless mouth ulcers, although these are usually early and short-lived (4).

The therapeutic approach for ReA involves treating the underlying infection with antibiotics, managing arthritis with nonsteroidal anti-inflammatory drugs (NSAIDs) or traditional DMARDs, and addressing extra-articular manifestations with topical or systemic medications. For severe KB and lesions, biologics may also be utilized. According to the 2014 European guidelines, patients with mild skin lesions or oral ulcers in ReA may not require immediate treatment. Mild to moderate KB can be managed with topical corticosteroids containing salicylic acid derivatives, while severe cases may necessitate systemic therapies like methotrexate or TNF inhibitors (6).

The pathogenesis of inflammatory skin diseases is intricately regulated by the dynamic interplay between cytokines, immune cells, and tissue cells. The JAK-STAT signaling pathway assumes a crucial role in this intricate network. The JAK family, comprising JAK1, JAK2, JAK3, and tyrosine kinase(TYK) 2, activates STATs through autophosphorylation. A multitude of cytokines heavily rely on the JAK-STAT pathway for their signaling, including IFN-α/β, IFN-γ, interleukins (IL-2, IL-4, IL-7, IL-9, IL-15, and IL-21) that share the IL-2 receptor common γ chain, IL-5, IL-6, IL-12, IL-13, and IL-23. Notably, while TNF-α, IL-1, and IL-17 do not directly engage the JAK-STAT pathway, the use of JAK inhibitors can indirectly impede upstream STAT-dependent cytokines (e.g., IL-23), thereby attenuating downstream cytokines (e.g., IL-17) (7).

Tofacitinib demonstrates a marked preference for inhibiting signaling pathways mediated by heterodimeric receptors associated with JAK1, and JAK3, with functional selectivity over JAK2-paired receptor (8). The drug’s mechanism of action involves inhibiting STAT phosphorylation and signal transduction, particularly targeting T helper (Th) 1, Th2, and Th17-related cytokines. These cytokines play a vital role in various skin diseases, such as IL-4 and IL-13, which contribute to keratinocyte damage and impaired skin barrier function (9). Conversely, elevated levels of IL-17 hinder skin repair (10), while overexpression of IL-31 is linked to pruritus (11). Additionally, IFN-γ mediates chronic inflammation and skin thickening (12). By suppressing pro-inflammatory cytokine gene transcription and interrupting T cell activation, tofacitinib effectively reduces inflammation and offers a rational approach for treating skin diseases by blocking JAK-STAT pathways and targeting related cytokines (13).

In this case, the patient presented with severe KB, and did not experience significant improvement despite the use of prednisone, adalimumab, and secukinumab. Therefore, we contend that TNF-α inhibitor and IL-17A inhibitor do not exert a therapeutic effect on the patient. Recent studies have reported that tofacitinib can be effective in treating patients with ReA accompanied by skin rashes. This became one of the major factors in our selection of tofacitinib as a therapeutic agent. A study on four patients with refractory ReA reported significant improvement in joint symptoms after one month of tofacitinib at 5 mg bid, including a case of severe plantar KB (14). A 45-year-old woman with plaque psoriasis and painful pustules on her palms and soles showed significant improvement in joint symptoms and lesion clearance after three months of tofacitinib (15). Another study involving six patients aged 42–58 with PPP demonstrated substantial improvement with tofacitinib, with all patients achieving at least a 50% reduction in palmoplantar pustular psoriasis area and severity index (PPPASI) after four weeks, and half experiencing more than an 80% reduction (16).

As an emerging class of drugs, recent studies have shown that JAK inhibitors, including tofacitinib, have demonstrated good efficacy in treating alopecia areata, atopic dermatitis, psoriasis, and vitiligo (7). In this case, the patient’s skin lesions significantly improved after treatment with tofacitinib, and no recurrence of similar rashes was observed during follow-ups. The successful treatment of this patient presents novel possibilities for utilizing JAK inhibitors in the management of challenging skin diseases.

## Data availability statement

The original contributions presented in the study are included in the article/**Supplementary Material**. Further inquiries can be directed to the corresponding author.

## Ethics statement

Written informed consent was obtained from the individual(s) for the publication of any potentially identifiable images or data included in this article.

## Author contributions

MM: Conceptualization, Data curation, Supervision, Writing – original draft. YL: Conceptualization, Formal Analysis, Writing – original draft. NK: Data curation, Formal Analysis, Writing – review & editing. YW: Supervision, Writing – review & editing. ZL: Formal Analysis, Supervision, Writing – review & editing. WH: Supervision, Writing – review & editing. SY: Conceptualization, Supervision, Writing – review & editing.
